# *N*-(2-Hydroxy­ethyl)-3,5-dinitro­benzamide

**DOI:** 10.1107/S1600536808019521

**Published:** 2008-07-05

**Authors:** Li He, Zhi-Gang Xu, Yan Jiang, Zhi-Hua Mao, An-Ping Deng

**Affiliations:** a College of Chemistry, Sichuan University, Chengdu 610064, People’s Republic of China; b Analytical and Testing Center, Sichuan University, Chengdu 610064, People’s Republic of China

## Abstract

The title compound, C_9_H_9_N_3_O_6_, was synthesized by the condensation of methyl 3,5-dinitro­benzoate and 2-amino­ethanol. The non-centrosymmetric space group results in the formation of pseudo-chiral helices in the crystal structure, which exhibits a layer packing structure involving intra­molecular N—H⋯O and O—H⋯O inter­actions.

## Related literature

For related literature, see: Lin & Smith (1981 ▶); Morehouse & McGuire (1959 ▶); Percec (1981 ▶, 1982 ▶); Walde (1962 ▶).
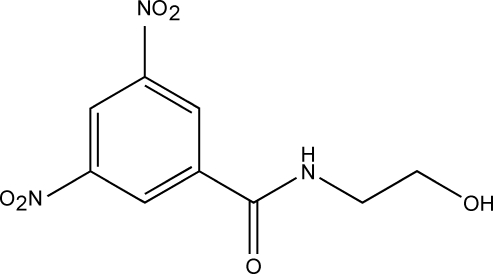


## Experimental

### 

#### Crystal data

C_9_H_9_N_3_O_6_
*M* *_r_* = 255.19
Orthorhombic, 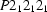

*a* = 6.514 (4) Å
*b* = 9.097 (3) Å
*c* = 18.177 (3) Å
*V* = 1077.1 (8) Å^3^
*Z* = 4
Mo *K*α radiation
μ = 0.14 mm^−1^
*T* = 294 (2) K
0.46 × 0.45 × 0.33 mm

#### Data collection

Enraf–Nonius CAD-4 diffractometer
Absorption correction: none
1124 measured reflections
1118 independent reflections
945 reflections with *I* > 2σ(*I*)
*R*_int_ = 0.015
3 standard reflections every 100 reflections intensity decay: 3.4%

#### Refinement

*R*[*F* ^2^ > 2σ(*F* ^2^)] = 0.035
*wR*(*F* ^2^) = 0.092
*S* = 1.10
1118 reflections
164 parameters
H-atom parameters constrained
Δρ_max_ = 0.14 e Å^−3^
Δρ_min_ = −0.18 e Å^−3^

### 

Data collection: *DIFRAC* (Gabe *et al.*, 1993 ▶); cell refinement: *DIFRAC*; data reduction: *NRCVAX* (Gabe *et al.*, 1989 ▶); program(s) used to solve structure: *SHELXS97* (Sheldrick, 2008 ▶); program(s) used to refine structure: *SHELXL97* (Sheldrick, 2008 ▶); molecular graphics: *ORTEP-3* (Farrugia, 1997 ▶) and *DIAMOND* (Brandenburg, 1998 ▶); software used to prepare material for publication: *SHELXL97*.

## Supplementary Material

Crystal structure: contains datablocks global, I. DOI: 10.1107/S1600536808019521/lx2057sup1.cif

Structure factors: contains datablocks I. DOI: 10.1107/S1600536808019521/lx2057Isup2.hkl

Additional supplementary materials:  crystallographic information; 3D view; checkCIF report

## Figures and Tables

**Table 1:** Hydrogen-bond geometry (Å, °)

*D*—H⋯*A*	*D*—H	H⋯*A*	*D*⋯*A*	*D*—H⋯*A*
O2—H2*W*⋯O1^i^	0.82	1.99	2.737 (3)	151
N1—H1*N*⋯O2^i^	0.86	2.12	2.935 (3)	158

Symmetry code: (i) 
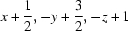
.

**Fig. 1.**
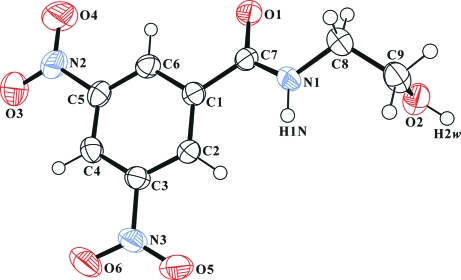
Molecular structure of title compound. Displacement ellipsoids are drawn at the 30% probability level.

**Fig. 2.**
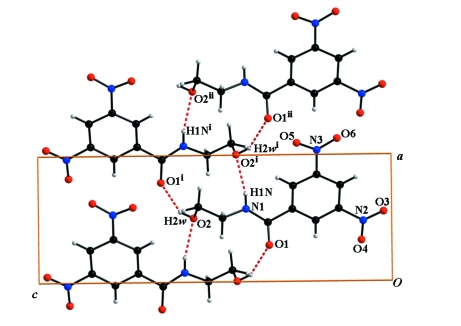
View of the structure projected on the ac plane. Hydrogen bonding shown as dashed lines. [Symmetry code: (i) x+1/2, -y+3/2, -z+1; (ii) x+1, y, z.]

## supplementary crystallographic information

### Comment

Non-ionic contrast agents, which are used in the field of intravascular and central nervous system visualization, are mostly complex molecules. However, the iodine in the molecule provides opacification to the *x*-rays and the remainder of the molecule provides the framework for transport of the iodine atoms. As a result, the structural arrangement of the molecule is very important in providing stability, solubility and biological safety in various organs (Lin & Smith, 1981). The title compound is an important intermediate in the synthesis of a variety of these molecules. It also can be used against coccidiosis and salmonella infection in poultry (Walde, 1962; Morehouse & McGuire, 1959).In addition, it plays an important role in the synthesis of copolymers (Percec, 1982; Percec, 1981). In this paper, we report the crystal structure of the title compound, N-(2-hydroxyethyl)-3,5-dinitrobenzamide (Fig. 1).

The title compound was crystallized in the non-centrosymmetric space group P212121 in spite of having no asymmetric carbon atom in the molecule. In the packing structure, an intermolecular O—H···O hydrogen bond leads to form pseudo-chiral helix about the 21 screw axis, propagating in the [100] direction. Non-centrosymmetric space group P212121 results in the formation of pseudo-chiral helix in the packing structure (Fig. 2). The crystal structure exhibits a layer packing structure with the intramolecular N—H···O and O—H···O hydrogen bonds (Fig. 2 and Table 1; symmetry code as in Fig. 2). On the other hand, adjacent molecules are linked into chains through van der Waals force to stabilize the crystal structure.

### Experimental

A mixture of methyl 3,5-dinitrobenzoate (5.65 g, 0.025 mol) and 50% aqueous 2-aminoethanol (30.5 g, 0.5 mol) was stirred for 10 h at room temperture. Then 30 ml water was added and the crystalline product was collected. Recrystallization of the crude product from ethanol gave N-(2-hydroxyethyl)-3,5-dinitrobenzamide (m.p. 416-417 K) (Lin & Smith, 1981). Single crystals of the title compound were obtained and used for X-ray diffraction studies at room temperature.

### Refinement

All H atoms were placed in idealized positions {C—H = 0.93 Å% (aromatic); C—H = 0.97 Å% (methylene); N—H = 0.86 Å%; O—H = 0.82 Å%} and refined as riding, with *U*_iso_(H) = 1.2*U*_eq_(C, N) or 1.5*U*_eq_(O). Friedel pairs were merged at final refinement.

### Crystal data

**Table:**

C_9_H_9_N_3_O_6_	*D*_x_ = 1.574 Mg m^−^^3^
*M**_r_* = 255.19	Melting point = 416–417 K
Orthorhombic, *P*2_1_2_1_2_1_	Mo *K*α radiation λ = 0.71073 Å
Hall symbol: p 2ac 2ab	Cell parameters from 24 reflections
*a* = 6.514 (4) Å	θ = 4.5–7.8º
*b* = 9.097 (3) Å	µ = 0.14 mm^−^^1^
*c* = 18.177 (3) Å	*T* = 294 (2) K
*V* = 1077.1 (8) Å^3^	Block, colourless
*Z* = 4	0.46 × 0.45 × 0.33 mm
*F*_000_ = 528	

### Data collection

**Table:**

Enraf–Nonius CAD-4 diffractometer	*R*_int_ = 0.015
Radiation source: fine-focus sealed tube	θ_max_ = 25.0º
Monochromator: graphite	θ_min_ = 2.2º
*T* = 294(2) K	*h* = −3→7
ω/2θ scans	*k* = −4→10
Absorption correction: none	*l* = −10→21
1124 measured reflections	3 standard reflections
1118 independent reflections	every 100 reflections
945 reflections with *I* > 2σ(*I*)	intensity decay: 3.4%

### Refinement

**Table:**

Refinement on *F*^2^	Hydrogen site location: inferred from neighbouring sites
Least-squares matrix: full	H-atom parameters constrained
*R*[*F*^2^ > 2σ(*F*^2^)] = 0.035	*w* = 1/[σ^2^(*F*_o_^2^) + (0.0522*P*)^2^ + 0.1396*P*] where *P* = (*F*_o_^2^ + 2*F*_c_^2^)/3
*wR*(*F*^2^) = 0.092	(Δ/σ)_max_ < 0.001
*S* = 1.10	Δρ_max_ = 0.14 e Å^−^^3^
1118 reflections	Δρ_min_ = −0.18 e Å^−^^3^
164 parameters	Extinction correction: SHELXL97 (Sheldrick, 2008), Fc^*^=kFc[1+0.001xFc^2^λ^3^/sin(2θ)]^-1/4^
Primary atom site location: structure-invariant direct methods	Extinction coefficient: 0.219 (12)
Secondary atom site location: difference Fourier map	

### Special details

**Table:**

Geometry. All esds (except the esd in the dihedral angle between two l.s. planes) are estimated using the full covariance matrix. The cell esds are taken into account individually in the estimation of esds in distances, angles and torsion angles; correlations between esds in cell parameters are only used when they are defined by crystal symmetry. An approximate (isotropic) treatment of cell esds is used for estimating esds involving l.s. planes.
Refinement. Refinement of F^2^ against ALL reflections. The weighted R-factor wR and goodness of fit S are based on F^2^, conventional R-factors R are based on F, with F set to zero for negative F^2^. The threshold expression of F^2^ > 2sigma(F^2^) is used only for calculating R-factors(gt) etc. and is not relevant to the choice of reflections for refinement. R-factors based on F^2^ are statistically about twice as large as those based on F, and R- factors based on ALL data will be even larger.

### Fractional atomic coordinates and isotropic or equivalent isotropic displacement parameters (Å^2^)

**Table:**

	*x*	*y*	*z*	*U*_iso_*/*U*_eq_	
O1	0.2873 (3)	0.5989 (2)	0.34735 (10)	0.0471 (6)	
O2	0.5044 (3)	0.7755 (2)	0.56083 (10)	0.0476 (6)	
H2W	0.5535	0.8106	0.5985	0.071*	
O3	0.5495 (4)	0.6710 (3)	0.02304 (11)	0.0717 (8)	
O4	0.3233 (5)	0.5573 (4)	0.08631 (13)	0.1040 (12)	
O5	1.0931 (4)	0.9372 (3)	0.26737 (12)	0.0799 (9)	
O6	1.1272 (4)	0.8922 (3)	0.15286 (11)	0.0696 (8)	
N1	0.5752 (4)	0.6420 (2)	0.41241 (11)	0.0370 (6)	
H1N	0.6916	0.6865	0.4132	0.044*	
N2	0.4757 (5)	0.6333 (3)	0.08103 (13)	0.0535 (7)	
N3	1.0356 (4)	0.8822 (3)	0.21046 (13)	0.0464 (6)	
C1	0.5713 (4)	0.6903 (3)	0.28124 (14)	0.0316 (6)	
C2	0.7552 (4)	0.7679 (3)	0.27890 (13)	0.0332 (6)	
H2A	0.8186	0.7982	0.3222	0.040*	
C3	0.8421 (4)	0.7994 (3)	0.21153 (14)	0.0355 (6)	
C4	0.7576 (4)	0.7570 (3)	0.14550 (13)	0.0366 (7)	
H4	0.8202	0.7779	0.1007	0.044*	
C5	0.5739 (5)	0.6816 (3)	0.14983 (14)	0.0386 (7)	
C6	0.4801 (4)	0.6487 (3)	0.21553 (14)	0.0358 (6)	
H6	0.3556	0.5987	0.2159	0.043*	
C7	0.4681 (4)	0.6420 (3)	0.35076 (14)	0.0350 (7)	
C8	0.5042 (5)	0.5698 (3)	0.47915 (13)	0.0437 (7)	
H8A	0.5403	0.4664	0.4768	0.052*	
H8B	0.3557	0.5765	0.4815	0.052*	
C9	0.5928 (4)	0.6344 (3)	0.54759 (14)	0.0426 (7)	
H9A	0.5652	0.5700	0.5890	0.051*	
H9B	0.7404	0.6438	0.5424	0.051*	

### Atomic displacement parameters (Å^2^)

**Table:**

	*U*^11^	*U*^22^	*U*^33^	*U*^12^	*U*^13^	*U*^23^
O1	0.0326 (11)	0.0647 (13)	0.0441 (10)	−0.0133 (11)	0.0046 (9)	−0.0125 (10)
O2	0.0324 (10)	0.0700 (13)	0.0405 (10)	0.0039 (11)	−0.0015 (9)	−0.0121 (9)
O3	0.089 (2)	0.0951 (17)	0.0311 (11)	−0.0176 (17)	−0.0042 (13)	0.0000 (11)
O4	0.103 (2)	0.156 (3)	0.0536 (14)	−0.079 (2)	−0.0175 (15)	−0.0111 (17)
O5	0.0793 (19)	0.113 (2)	0.0478 (13)	−0.0607 (17)	−0.0059 (12)	0.0036 (13)
O6	0.0530 (14)	0.104 (2)	0.0523 (13)	−0.0241 (16)	0.0145 (12)	0.0089 (13)
N1	0.0305 (12)	0.0486 (13)	0.0319 (11)	−0.0083 (12)	0.0060 (10)	0.0000 (10)
N2	0.0617 (19)	0.0631 (16)	0.0356 (13)	−0.0109 (18)	−0.0113 (14)	−0.0048 (12)
N3	0.0415 (14)	0.0577 (14)	0.0400 (13)	−0.0152 (13)	0.0011 (13)	0.0105 (13)
C1	0.0287 (13)	0.0319 (12)	0.0341 (13)	0.0022 (12)	0.0013 (12)	−0.0024 (11)
C2	0.0334 (14)	0.0363 (13)	0.0300 (12)	−0.0038 (12)	−0.0027 (12)	−0.0013 (11)
C3	0.0320 (13)	0.0378 (13)	0.0367 (13)	−0.0040 (12)	0.0003 (13)	0.0023 (12)
C4	0.0415 (17)	0.0383 (14)	0.0300 (12)	0.0006 (14)	0.0033 (13)	0.0031 (11)
C5	0.0442 (17)	0.0414 (14)	0.0303 (13)	0.0005 (15)	−0.0081 (12)	−0.0026 (11)
C6	0.0281 (13)	0.0400 (13)	0.0391 (14)	−0.0011 (12)	−0.0031 (13)	−0.0039 (12)
C7	0.0310 (16)	0.0392 (15)	0.0346 (14)	−0.0051 (14)	0.0027 (12)	−0.0081 (11)
C8	0.0455 (16)	0.0502 (15)	0.0354 (13)	−0.0075 (15)	0.0068 (14)	0.0031 (12)
C9	0.0345 (15)	0.0576 (17)	0.0356 (13)	0.0019 (16)	0.0030 (12)	0.0081 (13)

### Geometric parameters (Å, °)

**Table:**

O1—C7	1.243 (4)	C1—C7	1.498 (4)
O2—C9	1.428 (3)	C2—C3	1.379 (3)
O2—H2W	0.8200	C2—H2A	0.9300
O3—N2	1.208 (3)	C3—C4	1.376 (4)
O4—N2	1.213 (4)	C4—C5	1.382 (4)
O5—N3	1.209 (3)	C4—H4	0.9300
O6—N3	1.209 (3)	C5—C6	1.374 (4)
N1—C7	1.320 (3)	C6—H6	0.9300
N1—C8	1.455 (3)	C8—C9	1.492 (4)
N1—H1N	0.8600	C8—H8A	0.9700
N2—C5	1.472 (4)	C8—H8B	0.9700
N3—C3	1.468 (3)	C9—H9A	0.9700
C1—C6	1.387 (3)	C9—H9B	0.9700
C1—C2	1.391 (4)		
			
C9—O2—H2W	109.5	C6—C5—C4	122.9 (2)
C7—N1—C8	122.7 (2)	C6—C5—N2	118.7 (3)
C7—N1—H1N	118.7	C4—C5—N2	118.4 (3)
C8—N1—H1N	118.7	C5—C6—C1	119.9 (2)
O3—N2—O4	123.8 (3)	C5—C6—H6	120.0
O3—N2—C5	119.0 (3)	C1—C6—H6	120.0
O4—N2—C5	117.3 (3)	O1—C7—N1	122.9 (3)
O6—N3—O5	123.9 (2)	O1—C7—C1	118.4 (2)
O6—N3—C3	118.3 (2)	N1—C7—C1	118.6 (2)
O5—N3—C3	117.8 (2)	N1—C8—C9	113.2 (2)
C6—C1—C2	118.8 (2)	N1—C8—H8A	108.9
C6—C1—C7	117.0 (2)	C9—C8—H8A	108.9
C2—C1—C7	124.2 (2)	N1—C8—H8B	108.9
C3—C2—C1	119.1 (2)	C9—C8—H8B	108.9
C3—C2—H2A	120.5	H8A—C8—H8B	107.7
C1—C2—H2A	120.5	O2—C9—C8	109.8 (2)
C4—C3—C2	123.5 (2)	O2—C9—H9A	109.7
C4—C3—N3	118.4 (2)	C8—C9—H9A	109.7
C2—C3—N3	118.1 (2)	O2—C9—H9B	109.7
C3—C4—C5	115.9 (2)	C8—C9—H9B	109.7
C3—C4—H4	122.1	H9A—C9—H9B	108.2
C5—C4—H4	122.1		
			
C6—C1—C2—C3	−0.8 (3)	O3—N2—C5—C4	−5.8 (4)
C7—C1—C2—C3	176.3 (2)	O4—N2—C5—C4	174.3 (3)
C1—C2—C3—C4	−0.6 (4)	C4—C5—C6—C1	−0.8 (4)
C1—C2—C3—N3	179.6 (2)	N2—C5—C6—C1	178.7 (2)
O6—N3—C3—C4	−10.1 (4)	C2—C1—C6—C5	1.4 (4)
O5—N3—C3—C4	169.5 (3)	C7—C1—C6—C5	−175.9 (3)
O6—N3—C3—C2	169.7 (3)	C8—N1—C7—O1	10.3 (4)
O5—N3—C3—C2	−10.7 (4)	C8—N1—C7—C1	−167.1 (2)
C2—C3—C4—C5	1.2 (4)	C6—C1—C7—O1	−16.4 (4)
N3—C3—C4—C5	−179.0 (2)	C2—C1—C7—O1	166.4 (2)
C3—C4—C5—C6	−0.6 (4)	C6—C1—C7—N1	161.2 (2)
C3—C4—C5—N2	180.0 (3)	C2—C1—C7—N1	−16.0 (4)
O3—N2—C5—C6	174.7 (3)	C7—N1—C8—C9	−154.7 (3)
O4—N2—C5—C6	−5.2 (4)	N1—C8—C9—O2	71.7 (3)

### Hydrogen-bond geometry (Å, °)

**Table:**

*D*—H···*A*	*D*—H	H···*A*	*D*···*A*	*D*—H···*A*
O2—H2W···O1^i^	0.82	1.99	2.737 (3)	151
N1—H1N···O2^i^	0.86	2.12	2.935 (3)	158

Symmetry codes: (i) *x*+1/2, −*y*+3/2, −*z*+1.

## References

[bb1] Brandenburg, K. (1998). *DIAMOND* Crystal Impact GbR, Bonn, Germany.

[bb2] Farrugia, L. J. (1997). *J. Appl. Cryst.* **30**, 565.

[bb3] Gabe, E. J., Le Page, Y., Charland, J.-P., Lee, F. L. & White, P. S. (1989). *J. Appl. Cryst.* **22**, 384–387.

[bb4] Gabe, E. J., White, P. S. & Enright, G. D. (1993). *DIFRAC* American Crystallographic Association, Pittsburgh meeting. Abstract PA104.

[bb5] Lin, Y. L. & Smith, K. R. (1981). US Patent 4 284 620.

[bb6] Morehouse, N. F. & McGuire, W. C. (1959). *Poult. Sci.* **38**, 410–423.

[bb7] Percec, V. (1981). *Polym. Bull.* **5**, 651–657.

[bb8] Percec, V. (1982). *Polym. Prep.* **23**, 301–302.

[bb9] Sheldrick, G. M. (2008). *Acta Cryst.* A**64**, 112–122. 10.1107/S0108767307043930 18156677

[bb10] Walde, A. W. (1962). US Patent 3 015 606.

